# Quantitative assessment of CD44 genetic variants and cancer susceptibility in Asians: a meta-analysis

**DOI:** 10.18632/oncotarget.10951

**Published:** 2016-07-29

**Authors:** Vishal Chandra, Yun-Mi Lee, Usha Gupta, Balraj Mittal, Jong Joo Kim, Rajani Rai

**Affiliations:** ^1^ Department of Biosciences, Integral University, Lucknow, UP, India; ^2^ Stephenson Cancer Center (SCC), University of Oklahoma Health Sciences Center (OUHSC), Oklahoma City, OK, USA; ^3^ School of Biotechnology, Yeungnam University, Gyeongsan, Gyeongbuk, Korea; ^4^ Sanjay Gandhi Post Graduate Institute of Medical Sciences (SGPGIMS), Lucknow, India

**Keywords:** CD44, single nucleotide polymorphisms (SNP), meta-analysis, cancer, susceptibility

## Abstract

CD44 is a well-established cancer stem cell marker playing a crucial role in tumor metastasis, recurrence and chemo-resistance. Genetic variants of *CD44* have been shown to be associated with susceptibility to various cancers; however, the results are confounding. Hence, we performed a meta-analysis to clarify these associations more accurately. Overall, rs13347 (T *vs*. C: OR=1.30, *p*=<0.004, *p*_corr_=0.032; CT *vs*. CC: OR=1.29, p=0.015, *p*_corr_=0.047; TT *vs*. CC: OR=1.77, *p*=<0.000, *p*_corr_=0.018; CT+TT *vs*. CC: OR=1.34, *p*=<0.009, *p*_corr_=0.041) and rs187115 (GG *vs*. AA: OR=2.34, *p*=<0.000, *p*_corr_=0.025; AG *vs*. AA: OR=1.59, *p*=<0.000, *p*_corr_=0.038; G *vs*. A allele OR=1.56, *p*=0.000, *p*_corr_=0.05; AG+GG *vs*. AA: OR=1.63, *p*=<0.000, *p*_corr_=0.013) polymorphisms were found to significantly increase the cancer risk in Asians. On the other hand, rs11821102 was found to confer low risk (A *vs*. G: OR=0.87, *p*=<0.027, *p*_corr_=0.04; AG *vs*. GG: OR=0.85, *p*=<0.017, *p*_corr_=0.01; AG+AA *vs*. GG: OR=0.86, *p*=<0.020, *p*_corr_=0.02). Based on our analysis, we suggest significant role of *CD44* variants (rs13347, rs187115 and rs11821102) in modulating individual's cancer susceptibility in Asians. Therefore, these variants may be used as predictive genetic biomarkers for cancer predisposition in Asian populations. However, more comprehensive studies involving other cancers and/or populations, haplotypes, gene-gene and gene-environment interactions are necessary to delineate the role of these variants in conferring cancer risk.

## INTRODUCTION

Cancer, which is an extremely complex and multifaceted disease involving multiple steps, is a leading cause of death worldwide. During last decades, considerable advancements have taken place in the development of better therapeutic interventions for cancer; however, chemo-resistance and disease recurrence have resulted in minimal disease outcome and poor survival rates [1]. Cancer stem cells (CSCs) are a small population of cells within a tumor and play a crucial role in cancer progression and recurrence. Because of their ability for self-renewal, they may initiate tumor growth and promote metastasis; thereby leading to aggressive forms of the disease. [2-6]. Hence, CSCs represent the most attractive and promising targets in clinical oncology [7].

Cluster of differentiation (CD) 44, a well-recognized CSC marker [8, 9], is a multistructural and multifunctionaltransmembrane glycoprotein that belongs to a family of cell adhesion receptors and is widely expressed in most mammalian cells [10, 11]. The gene for CD44 is complex (aprox 50 Kb), located on human chromosome 11p13, comprising 20 exons out of which 10 exons (exon 6-15) are involved in alternative splicing of *CD44* to generate many standard (CD44s) and variant isoforms (CD44v) of varying sizes [12, 13]. Although CD44 is the major receptor for hyaluronan (HA), the main component of the extracellular matrix, it can also bind with MMPs, collagens and osteopontin. It is involved in maintenance of cell-cell/extracellular matrix (ECM) interactions, cell adhesion, cell trafficking and migration etc. [14-17]. In addition, it mediates multiple vital biological processes such as angiogenesis, cell proliferation, cell differentiation and presentation ofcytokines,chemokines and growth factors to the corresponding receptors, docking of proteases as well as cell survival signaling that are closely associated with neoplastic transformation and tumor progression [18-20].

Several evidences have firmly established the role of CD44 in cell differentiation, epithelial -mesenchymal transition (EMT), invasion and metastatic spread in various human cancers [21-25]. In addition, CD44 aberrations have been shown to confer apoptosis resistance [26]. It was found to act like a tumor promoter in some cancers while it functioned as a tumor-suppressor in others [27]. Increased or decreased expression ofmoleculeshas been reported in various cancers and shown to be associated with increased tumor aggressiveness, metastasis, early tumor recurrence and chemo- or radio- resistance, as well as poor prognosis [28-31]. Further, CD44 targeting by monoclonal antibodies and blocking peptides has been established as a promising therapeutic approach for cancer [32-34].

Considering the important role of *CD44* in carcinogenesis, several studies have explored the role of genetic variants of *CD44* in cancer susceptibility, prognosis and chemotherapeutic response in various human cancers [35-38]. However, the results are controversial and the power of each study was restricted due to low sample size, necessitating further clarification of its role in cancer predisposition. Hence, we performed a meta-analysis of all eligible case-control studies to better interpret the associations between common SNPs of the *CD44* gene (rs13347 C>T, rs10836347 C>T, rs11821102 G>A, rs713330 T>C, rs187115 T>C) and cancer risk.

## RESULTS

According to the search strategies mentioned above, we found a total of 13 case-control studies investigating the association of *CD44* polymorphisms (rs13347 C>T, rs10836347 C>T, rs11821102 G>A, rs713330 T>C, rs187115 T>C) with cancer susceptibility [36, 38-49]. However, the study by Qiu et al. [49] in Chinese gastric patients lacked genotyping details for each of the studied SNP, hence excluded. Therefore, we included only 12 potential case-control studies in the present meta-analysis and the characteristics of each eligible study are presented in Table 1. Since all studies were performed in Asian populations and are limited for cancer types, we performed subgroup analysis only based on study design (population based; PB, hospital based; HB), cancer types (gastrointestinal cancer; GIC, Head and neck cancer; HNC, and other cancer) and genotyping methods (Taqman or others).

**Table 1:** Studies Included in CD44 Meta-Analysis

Author	Country /Ethnicity	Cancer type	Design	Case total	WW	WV	VV	Control total	WW	WV	VV	pHWE	Genotyping Method
***CD44* rs13347**													
Jiang et al., 2012	China/Asian	BRC	PB	1853	813	850	190	1992	1146	727	119	0.7949	MassArray
Tulsyan et al., 2013	India/Asian	BRC	HP	258	191	60	7	241	178	57	6	0.5773	Taqman
Xiao et al., 2013	China/Asian	NPC	PB	906	386	418	102	943	606	297	40	0.6367	MassArray
Sharma et al., 2014	India/Asian	GBC	HP	405	293	104	8	200	154	42	4	0.5716	Taqman
Chou et al., 2014	Taiwan/Asian	OC	HP	599	287	262	50	561	295	223	43	0.9241	Taqman
Chou et al., 2014	Taiwan/Asian	HCC	HP	203	110	72	21	561	295	223	43	0.9241	Taqman
Lou et al., 2014	China/Asian	NPC	HP	272	104	126	42	489	288	174	27	0.9147	Sequencing
Weng et al., 2015	Taiwan/Asian	BC	HP	275	138	111	26	275	143	117	15	0.1527	Taqman
Wu et al., 2015	China/Asian	CRC	PB	946	416	441	89	989	578	348	63	0.2788	MALDI-TOF
Wu et al., 2015	China/Asian	AML	PB	421	163	196	62	461	254	171	36	0.3398	MALDI-TOF
Liu et al., 2015	China/Asian	NSCLC	HP	234	179	51	4	468	337	121	10	0.8227	Taqman
Verma et al., 2016	India/Asian	BC	HP	240	152	73	15	270	140	104	26	0.30443	Taqman
***CD44* rs11821102**													
Jiang et al., 2012	China/Asian	BRC	PB	1049	912	125	12	1157	997	151	9	0.2193	MassArray
Xiao et al., 2013	China/Asian	NPC	PB	906	796	100	10	943	805	129	9	0.1383	MassArray
Chou et al., 2014	Taiwan/Asian	OC	HP	599	531	63	5	561	481	75	5	0.283	Taqman
Chou et al., 2014	Taiwan/Asian	HCC	HP	203	173	29	1	561	481	75	5	0.283	Taqman
Lou et al., 2014	China/Asian	NPC	HP	280	252	27	1	496	439	54	3	0.3489	Sequencing
Weng et al., 2015	Taiwan/Asian	BC(TCC)	HP	275	234	39	2	275	222	50	3	0.9217	Taqman
Wu et al., 2015	China/Asian	CRC	PB	946	815	119	12	989	843	131	15	**0.0003**	MALDI-TOF
Wu et al., 2015	China/Asian	AML	PB	421	370	50	1	461	398	59	4	0.2792	MALDI-TOF
***CD44* rs10836347**													
Jiang et al., 2012	China/Asian	BRC	PB	1049	906	139	4	1157	995	156	6	0.9657	MassArray
Xiao et al., 2013	China/Asian	NPC	PB	906	785	118	3	943	792	147	4	0.3064	MassArray
Chou et al., 2014	Taiwan/Asian	OC	HP	599	522	73	4	561	487	69	5	0.1524	Taqman
Chou et al., 2014	Taiwan/Asian	HCC	HP	203	180	23	0	561	487	69	5	0.1524	Taqman
Lou et al., 2014	China/Asian	NPC	HP	278	249	27	2	495	438	55	2	0.8462	Sequencing
Wu et al., 2015	China/Asian	CRC	PB	946	821	120	5	989	851	129	9	0.102	MALDI-TOF
Wu et al., 2015	China/Asian	AML	PB	421	364	55	2	461	404	55	2	0.9304	MALDI-TOF
***CD44* rs713330**													
Jiang et al., 2012	China/Asian	BRC	PB	1049	865	172	12	1157	950	194	13	0.3851	MassArray
Xiao et al., 2013	China/Asian	NPC	PB	906	732	164	10	943	751	180	12	0.7441	MassArray
Chou et al., 2014	Taiwan/Asian	OC	HP	599	507	88	4	561	467	86	8	0.0857	Taqman
Chou et al., 2014	Taiwan/Asian	HCC	HP	203	167	36	0	561	467	86	8	0.0857	Taqman
Weng et al., 2015	Taiwan/Asian	BC(TCC)	HP	275	231	42	2	275	223	49	3	0.8669	Taqman
Wu et al., 2015	China/Asian	AML	PB	421	341	74	6	461	371	87	3	0.3854	MALDI-TOF
***CD44* rs187115**													
Sharma et al., 2014	India/Asian	GBC	HP	405	248	126	31	200	125	61	14	0.0939	Taqman
Chou et al., 2014	Taiwan/Asian	OC	HP	599	336	227	36	561	403	143	15	0.5904	Taqman
Chou et al., 2014	Taiwan/Asian	HCC	HP	203	123	66	14	561	403	143	15	0.5904	Taqman
Weng et al., 2015	Taiwan/Asian	BC(TCC)	HP	275	178	87	10	275	204	68	3	0.3056	Taqman
Liu et al., 2015	China/Asian	NSCLC	HP	234	133	86	15	468	336	119	13	0.5322	Taqman
Verma et al., 2016	India/Asian	BC	HP	240	101	97	42	270	127	101	42	**0.0053**	Taqman

BRC- Breast Cancer, NPC- Nasopharyngeal Carcinoma, GBC- Gallbladder Cancer, OC- Oral Cancer, HCC- Hepatocellular Carcinoma, BC (TCC)- Bladder Cancer (Transitional Cell Carcinoma), CRC- Colorectal Cancer, AML- Acute Myeloid Leukemia, NSCLC- Non-Small Cell Lung Cancer, MALDI-TOF-MS- Matrix Assisted Laser Desorption/Ionization Time of Flight, W - Wild allele, V - Variant allele.

### Quality assessment

According to the Newcastle-Ottawa quality assessment scale (NOS), the quality of all recruited case-control studies and their total quality scores are summarized in Table 2. The quality scores ranged from 6 to 8 and the average score of case-control studies was 7.08. Thus, our NOS results indicated that most of these studies (9) in our meta-analysis were of high quality (NOS score 7 or 8) and only three studies with NOS score of 6 were classified into intermediate quality.

**Table 2:** Newcastle-Ottawa Scale Based Quality Assessment of Studies Included in CD44 Meta-Analysis

Author Name	Selection				Comparability		Exposure			Total scores
	1	2	3	4	1	2	1	2	3	
Jiang et al., 2012	*	*	*	*	*	*	*	*	-	8
Tulsyan et al., 2013	*	*	-	*	*	*	*	*	-	7
Xiao et al., 2013	*	*	*	*	*	*	*	*	-	8
Sharma et al., 2014	*	*	-	*	*	*	*	*	-	7
Chou et al., 2014	*	*	-	*	*	*	*	*	-	7
Chou et al., 2014	*	*	-	*	*	*	*	*	-	7
Lou et al., 2014	*	*	-	*	*		*	*	-	6
Weng et al., 2015	*	*	-	*	*		*	*	-	6
Wu et al., 2015	*	*	*	*	*	*	*	*	-	8
Wu et al., 2015	*	*	*	*	*		*	*	-	8
Liu et al., 2015	*	*	-	*	*	*	*	*	-	6
Verma et al., 2016	*	*	-	*	*	*	*	*	-	7

**Selection: (1)** Adequate definition of cases (yes, with independent validation, one star); **(2)** representativeness of the cases (if yes, one star); **(3)** selection of controls (one star for community controls,); **(4)** definition of controls (if no history of disease, one star). **Comparability:** comparability of cases and controls on the basis of design or analysis: **(1)** ethnicity matched (if yes, one star); **(2)** analysis age adjusted (if yes, one star). **Exposure: (1)** ascertainment of exposure (if in reliable method, one star); **(2)**, same method of ascertainment for cases and controls (if yes, one star); **(3)** non-response rate (same rate for both group, one star).

### *CD44* rs13347

For *CD44* rs13347 meta-analysis, a total of 12 articles [36, 38-48] with 6612 multiple cancer cases and 7450 controls were found to be eligible. The minor allele frequency (MAF) for rs13347 polymorphism varied from 13-29%. Overall, the variant allele and all genotypic models having at least one variant allele of rs13347 polymorphism were found to significantly increase the overall cancer risk compared with the wild allele/genotype. (T *vs*. C: OR = 1.30, 95% CI = 1.09-1.56, *p* = <0.004; CT *vs*. CC: OR = 1.29, 95% CI = 1.05-1.58, *p* = 0.015; TT *vs*. CC: OR = 1.77, 95% CI = 1.28-2.44, *p* = <0.000, CT+TT *vs*. CC: OR = 1.34, 95% CI = 1.08-1.67, *p* = <0.009, Table 3, Figure 2. and 3.). For this SNP, we used random effect model as the present meta-analysis revealed significant heterogeneity in all genotypic models. The removal of Lou et al. [39], Jiang et al. [42], Wu et al. [41, 48], and Xiao et al. [40] were found to remove heterogeneity for hetero as well as variant models (CT *vs*. CC: ph = 0.085, I^2^ = 46.057; TT *vs*. CC: ph = 0.288, I^2^ = 1o.17) while removal of above studies together with Verma et al. [47] was found to remove heterogeneity at allele level and in dominant model (T *vs*. C: ph = 0.576, I^2^ = 0.000; CT+TT *vs*. CC: ph = 0.386, I^2^ = 4.764) However, it was found to significantly change the pooled results. In our sensitivity analysis, we did not find any obvious change in the corresponding pooled ORs after removing one study each time for a genetic model, thereby confirming reliability of our results.

**Table 3:** Meta-Analysis Result for *CD44* Polymorphism

Subgroup	*N*	Case/Control	V *vs*. W allele			VW *vs*. WW			VV *vs*. WW			VW+VV *vs*. WW		
			OR (95% CI)	p/_pcorr_	p_h_/I2	OR (95% CI)	p/pcorr	p_h_/I^2^	OR (95% CI)	p/p_corr_	p_h_/I^2^	OR (95% CI)	p/p_corr_	p_h_/I^2^
***CD44* rs13347**														
**Overall**	12	6612/7450	1.30 (1.09-1.56)	**0.004/ 0.032**	**0.000/ 89.625**	1.29 (1.05-1.58)	**0.015/ 0.047**	**0.000/ 85.803**	1.77 (1.28-2.44)	**0.000/ 0.018**	**0.000/ 77.90**	1.34 (1.08-1.67)	**0.009/ 0.041**	**0.000/ 88.919**
**HB subgroup**	8	2486/3065	1.09 (0.86-1.38)	0.483	**0.000/ 84.365**	1.05 (0.82-1.34)	’0.712	**0.000/ 74.741**	1.31 (0.80-2.15)	0.290	**0.000/ 74.534**	1.08 (0.82-1.42)	0.578	**0.000/ 81.751**
**PB subgroup**	4	4126/4385	1.76 (1.50-2.07)	0.000/ 0.029	**0.002/ 79.844**	1.80 (1.64-1.97)	**0.000/ 0.024**	0.115/ 49.370	2.58 (1.93-3.45)	**0.000/ 0.015**	**0.040/ 64.015**	1.94 (1.66-2.27)	**0.000/ 0.005**	**0.031/ 66.263**
**Taqman method**	7	2214/2576	0.99 (0.85-1.16)	0.930	**0.040/ 54.640**	0.98 (0.86-1.11)	0.729	0.085/ 46.057	1.11 (0.86-1.44)	0.429	0.288/ 18.617	0.97 (0.81-1.17)	0.751	**0.047/ 53.014**
**Other method**	5	4398/4874	1.81 (1.56-2.09)	**0.000/ 0.026**	**0.001/ 77.259**	1.81 (1.66-1.98)	**0.000/ 0.021**	0.175/ 36.989	2.79 (2.09-3.74)	**0.000/ 0.012**	**0.017/ 66.908**	1.99 (1.72-2.30)	**0.000/ 0.003**	**0.034/ 61.608**
**GIC**	3	1554/1750	1.27 (0.96-1.68)	0.097	**0.017/ 75.363**	1.28 (0.81-2.01)	0.289	**0.002/ 84.534**	1.71 (1.28-2.28)	**0.000/ 0.009**	0.354/ 3.699	1.31 (0.86-2.00)	0.208	**0.002/ 84.046**
**HNC**	3	1777/1993	1.70 (1.14-2.55)	**0.010/ 0.044**	**0.000/ 93.178**	1.75 (1.18-2.59)	**0.005/ 0.035**	**0.001/ 86.777**	2.73 (1.20-6.23)	**0.017/ 0.05**	**0.000/ 90.086**	1.89 (1.19-2.99)	**0.007/ 0.038**	**0.000/ 91.311**
**Other**	6	3281/3707	1.14 (0.83-1.56)	0.411	**0.000/ 91.233**	1.09 (0.78-1.53)	0.624	**0.000/ 87.511**	1.44 (0.87-2.38)	0.152	**0.001/ 76.247**	1.12 (0.78-1.61)	0.540	**0.000/ 90.185**
***CD44* rs11821102**														
**Overall**	7	3733/4454	0.87 (0.77-0.99)	**0.027/ 0.04**	0.891/ 0.000	0.85 (0.74-0.97	**0.017/ 0.01**	0.880/ 0.000	0.98 (0.60-1.61)	0.95	0.817/ 0.000	0.86 (0.75-0.98)	**0.020/ 0.02**	0.895/ 0.000
**HB subgroup**	4	1357/1893	0.83 (0.68-1.02)	0.072	0.771/ 0.000	0. 84 (0.68-1.04)	0.106	0.636/ 0.000	0.73 (0.31-1.71)	0.463	0.971/ 0.000	0.83 (0.67-1.02)	0.082	0.692/ 0.000
**PB subgroup**	3	2376/2561	0.90 (0.77-1.05)	0.163	0.655/ 0.000	0.86 (0.73-1.02)	0.080	0.719/ 0.000	1.14 (0.63-2.09)	0.436	0.373/ 0.000	0.87 (0.74-1.03)	0.109	0.723/ 0.000
**Taqman method**	3	1077/1397	0.83 (0.67-1.03)	0.097	0.572/ 0.000	0.83 (0.65-1.05)	0.126	0.433/ 0.000	0.75 (0.30-1.90)	0.548	0.906/ 0.000	0.82 (0.65-1.04)	0.105	0.488/ 0.000
**Other method**	4	2656/3057	0.89 (0.77-1.03)	0.122	0.826/ 0.000	0.86 (0.73-1.01)	0.066	0.882/ 0.000	1.09 (0.61-1.96)	0.764	0.514/ 0.000	0.87 (0.75-1.02)	0.085	0.884/ 0.000
**HNC**	3	1785/2000	0.83 (0.69-0.99)	**0.038/ 0.05**	0.954/ 0.000	0.79 (0.65-0.97)	**0.022/ 0.03**	0.905/ 0.000	0.99 (0.49-1.98)	0.970	0.858/ 0.000	0.80 (0.66-0.97)	0.026	0.939/ 0.000
**Other + GIC**	4	1948/2454	0.91 (0.76-1.07)	0.270	0.671/ 0.000	0.90 (0.75-1.08)	0.257	0.735/ .0.000	0.98 (0.49-1.97)	0.955	0.453/ 0.000	0.90 (0.76-1.08)	0.255	0.723/ 0.000
***CD44* rs10836347**														
Overall	7	4402/5167	0.93 (0.82-1.04)	0.192	0.844/ 0.000	0.94 (0.83-1.06)	0.291	0.894/ 0.000	0.74 (0.42-1.30)	0.297	0.948/ 0.000	0.93 (0.82-1.04)	0.206	0.896/ 0.000
HB subgroup	3	1080/1617	0.93 (0.72-1.21)	0.606	0.662/ 0.000	0.93 (0.73-1.20)	0.581	0.899/ 0.000	0.82 (0.29-2.29)	0.705	0.532/ 0.000	0.92 (0.72-1.17)	0.492	0.890/ 0.000
PB subgroup	4	3322/3550	0.92 (0.81-1.05)	0.230	0.598/ 0.000	0.94 (0.82-1.08)	0.367	0.563/ 0.000	0.71 (0.36-1.40)	0.317	0.951/ 0.000	0.93 (0.82-1.07)	0.287	0.569/ 0.000
Taqman method	2	802/ 1122	0.94 (0.68-1.29)	0.680	0.364/ 0.000	0.96 (0.72-1.28)	0.771	0.773 /0.000	0.62 (0.19-2.05)	0.430	0.494/ 0.000	0.93 (0.70-1.23)	0.599	0.641/ 0.000
Other method	5	3600/4045	0.92 (0.82-1.05)	0.215	0.757/ 0.000	0.93 (0.82-1.07)	0.303	0.708/ 0.000	0.78 (0.41-1.48)	0.447	0.897/ 0.000	0.93 (0.81-1.06)	0.250	0.728/ 0.000
GIC	2	481/ 1056	0.86 (0.62-1.20)	0.382	0.629/ 0.000	0.88 (0.62-1.25)	0.480	0.903/ 0.000	0.95 (0.19-4.82)	0.947	0.271/ 0.000	0.87 (0.62-1.23)	0.422	0.859/ 0.000
HNC	3	1926/1965	0.93 (0.77-1.12)	0.418	0.334/ 8.802	0.92 (0.76-1.10)	0.356	0.385/ 0.000	0.81 (0.34-1.97)	0.646	0.941/ 0.000	0.91 (0.76-1.10)	0.323	0.383/ 0.000
Other	2	1995/2146	0.94 (0.80-1.11)	0.483	0.787/ 0.000	0.97 (0.81-1.16)	0.757	0.936/ 0.000	0.64 (0.28-1.46)	0.289	0.779/ 0.000	0.96 (0.80-1.14)	0.612	0.860/ 0.000
***CD44* rs713330**														
Overall	6	3453/3958	0.94 (0.84-1.05)	0.289	0.953/ 0.000	0.96 (0.85-1.08)	0.464	0.929/ 0.000	0.86 (0.54-1.37)	0.532	0.509/ 0.000	0.95 (0.84-1.07)	0.361	0.965/ 0.000
HB subgroup	3	1077/1397	0.88 (0.72-1.08)	0.236	0.837/ 0.000	0.97 (0.76-1.21)	0.781	0.535/ 0.000	0.45 (0.18-1.16)	0.099	0.729/ 0.000	0.92 (0.74-1.15)	0.464	0.674/ 0.000
PB subgroup	3	2376/2561	0.97 (0.85-1.10)	0.620	0.892/ 0.000	0.95 (0.82-1.10)	0.488	0.958/ 0.000	1.06 (0.62-1.81)	0.832	0.526/ 0.000	0.96 (0.83-1.10)	0.539	0.953/ 0.000
***CD44* rs187115**														
Overall	5	1716/2065	1.56 (1.29-1.90)	**0.000/ 0.05**	**0.035/ 61.402**	1.59 (1.37-1.84)	**0.000/ 0.038**	0.102/ 48.313	2.34 (1.67-3.27)	**0.000/ 0.025**	0.165/ 38.482	1.63 (1.30-2.03)	**0.000/ 0.013**	**0.048/ 58.222**

Significant associations are shown in bold, Ph- p value of Q test for heterogeneity, OR- Odds ratio, CI- Confidence interval, W-Wild allele, V- Variant allele, HB- Hospital based, PB-Population based, GIC- Gastrointestinal cancer (including - Gallbladder Cancer/GBC, Hepatocellular Carcinoma/HCC, Colorectal Cancer/CRC), HNC- Head and neck cancers (including Nasopharyngeal Carcinoma/NPC, Oral Cancer/OC)

**Figure 1:**
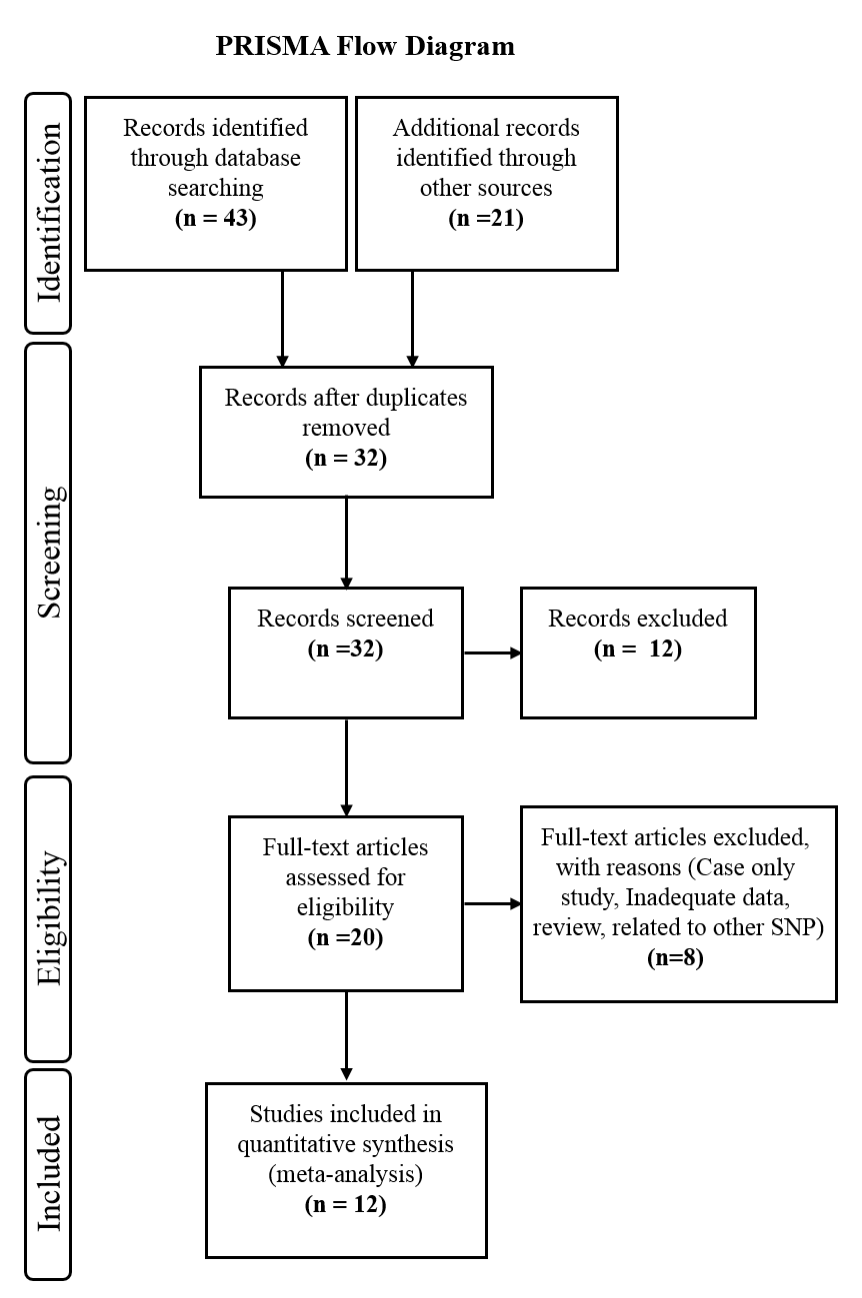
Flow diagram of the study selection process

**Figure 2:**
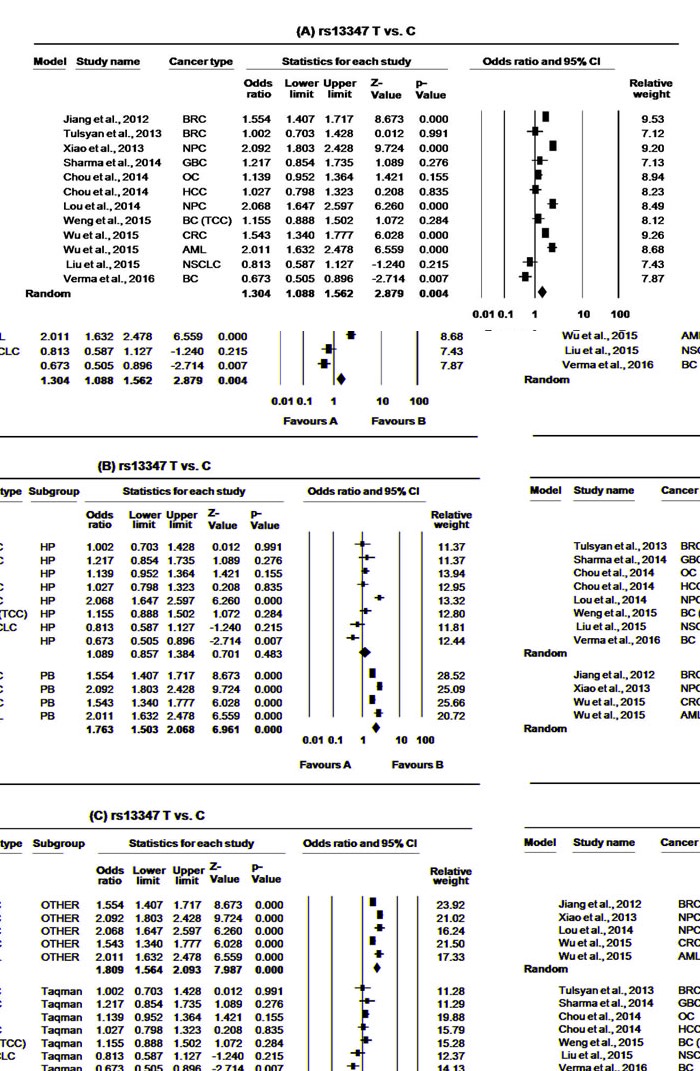
Forest plots for meta-analysis of *CD44* rs13347 polymorphism T *vs*. C **A.** Overall, **B.** stratification on the basis of study design and **C.** stratification on the basis of genotyping method. For each study, the estimates of OR and 95% CI were plotted with squares and horizontal lines. The size of the square points is the relative weight of the respective study. Diamonds indicate the pooled OR and its 95% CI.

**Figure 3:**
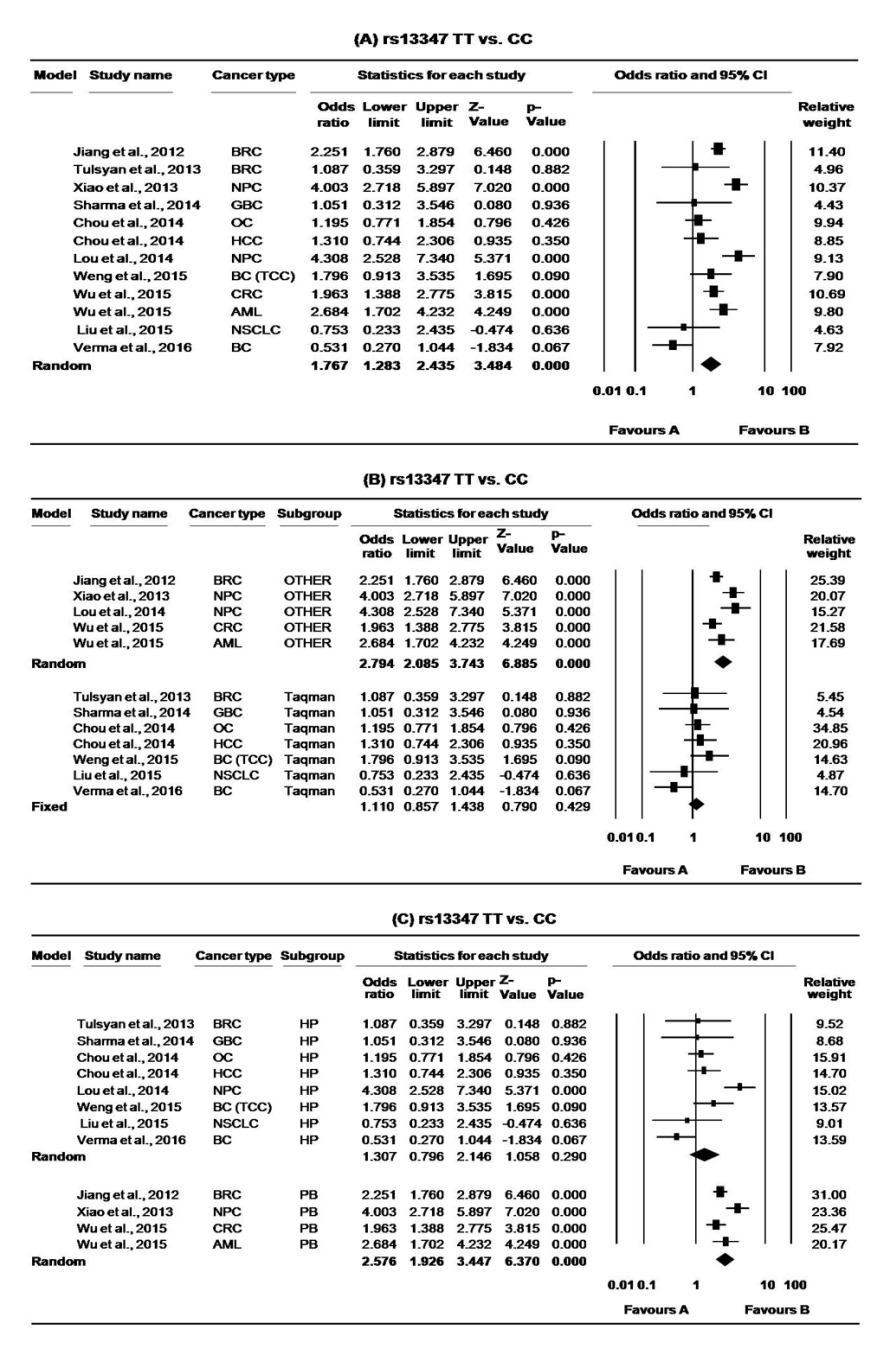
Forest plots for meta-analysis of *CD44* rs13347 polymorphism TT *vs*. CC **A.** Overall, **B.** stratification on the basis of genotyping method and **C.** stratification on the basis of study design. For each study, the estimates of OR and 95% CI were plotted with squares and horizontal lines. The size of the square points is the relative weight of the respective study. Diamonds indicate the pooled OR and its 95% CI.

In subgroup analysis based on study design and genotyping method (Taqman and/or other), the significant association was limited only to population based studies (T *vs*. C: OR = 1.76, 95% CI = 1.50-2.07, *p* = <0.000; CT *vs*. CC: OR = 1.80, 95% CI = 1.64-1.97, *p* = 0.000; TT *vs*. CC: OR = 2.58, 95% CI = 1.93-3.45, *p* = <0.000, CT+TT *vs*. CC: OR = 1.94, 95% CI = 1.66-2.27, *p* = <0.000, Figure 2. and 3.) as well as for other genotypic (non-Taqman) methods (T *vs*. C: OR = 1.81, 95% CI = 1.56-2.09, *p* = <0.000; CT *vs*. CC: OR = 1.81, 95% CI = 1.66-1.98, *p* = 0.000; TT *vs*. CC: OR = 2.79, 95% CI = 2.09-3.74, *p* = <0.000, CT+TT vs. CC: OR = 1.99, 95% CI = 1.72-2.30, *p* = <0.000, Figure 2. and 3.). Further, we also performed subgroup analysis on the basis of cancer types and the association was limited to HNCs (T *vs*. C: OR = 1.70, 95% CI = 1.14-2.55, *p* = <0.010; CT *vs*. CC: OR = 1.75, 95% CI = 1.18-2.59, *p* = 0.005; TT *vs*. CC: OR = 2.73, 95% CI = 1.20-6.23, *p* = <0.017, CT+TT *vs*. CC: OR = 1.89, 95% CI = 1.19-2.99, *p* = <0.007, Table 3).

### *CD44* rs11821102

**Figure 4:**
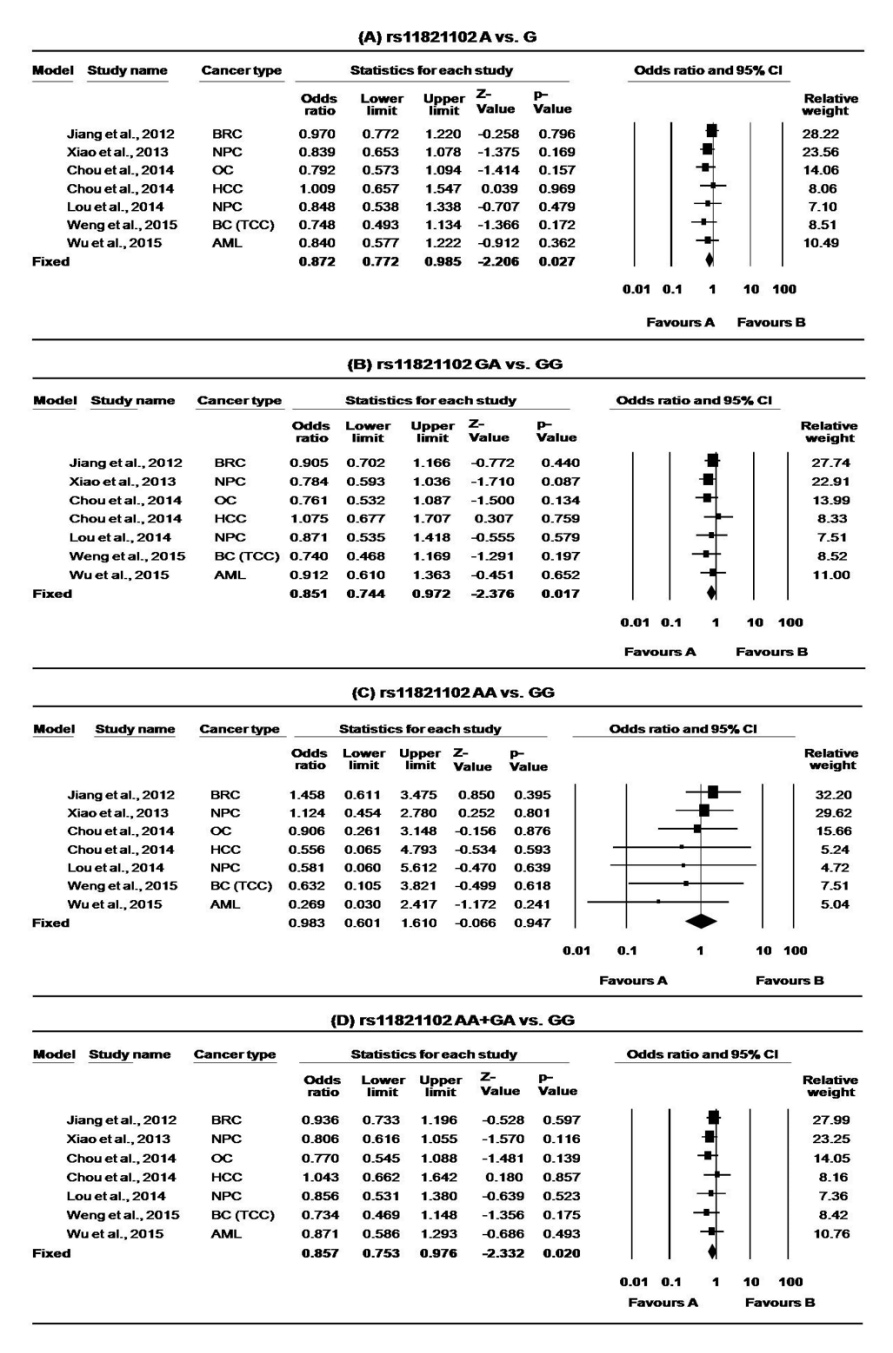
Forest plots for meta-analysis of *CD44* rs11821102 polymorphism **A.** A *vs*. G, **B.** GA *vs*. GG, **C.** AA *vs*. GG and **D.** AA+AG *vs*. GG and overall cancer risk. For each study, the estimates of OR and 95% CI were plotted with square and horizontal lines. The size of the square points is the relative weight of the respective study. Diamonds indicate the pooled OR and its 95% CI.

Among 11, only eight studies [36, 39-42, 45, 46, 48] investigated the association of rs11821102 polymorphism and cancer risk, however the study of Wu et al. [48] on CRC failed to follow the HWE in controls and was hence excluded. Thus, seven studies with 3733 multiple cancer cases and 4454 healthy controls were included for rs11821102 meta-analysis. The minor allele frequency (MAF) for rs11821102 SNP varied from 6-10% and overall it was found to reduce the risk of cancer in most of the genotypic models (A *vs*. G: OR = 0.87, 95% CI = 0.77-0.99, *p* = <0.027; AG *vs*. GG: OR = 0.85, 95% CI = 0.74-0.97, *p* = <0.017; AG+AA *vs*. GG: OR = 0.86, 95% CI = 0.75-0.98, *p* = <0.020, Table 3., Figure 4.) except for the variant genotype model (AA *vs*. GG: OR = 0.98, 95% CI = 0.60-1.61, *p* = 0.95, Table 3.). We did not encounter any significant heterogeneity in the selected studies. In sensitivity analysis, removal of two studies by Chou et al. [45] or Xiao et al. [40] was found to alter the corresponding statistical p value of association in hetero and dominant models while the removal of Weng et al. [36], Chou et al. [45] and Xiao et al. [40] was found to alter the pooled OR at allele level.

Further, in stratified analysis this association was lost in each subgroup except for HNC at allele level as well as at hetero genotype model (A *vs*. G: OR = 0.83, 95% CI = 0.69-0.99, *p* = <0.038; AG *vs*. GG: OR = 0.79, 95% CI = 0.65-0.97, *p* = <0.022;). This may be because the small sample size of the subgroup did not possess sufficient statistical power to detect a weak effect.

### *CD44* rs10836347

Among 11 studies, only seven (with a total of 4402 multiple cancer cases and 5167 controls) investigated the association of rs10836347 polymorphism in various cancers [39-42, 45, 46, 48]. The MAF for rs10836347 SNP varies from 6-8%. However, none of the genotypic combinations were found to affect the risk of overall cancer compared with the wild genotype (Table 3.). Our meta-analysis result was without any significant heterogeneity. The sensitivity analysis also confirmed the reliability of our result. Stratified analysis based on study design, cancer types and genotyping method did not modify the pooled result (Table 3.).).

### *CD44* rs713330

For rs713330, we found a total of six eligible studies with 3453 cancer cases and 3958 controls [36, 41, 42, 45, 46, 48]. The MAF for rs713330 polymorphism varies as 9-11% in controls. Overall, none of the genotypic combinations were found to affect the risk of overall cancer compared with the wild genotype (Table 3.). Our meta-analysis result was without any significant heterogeneity. The reliability of these results was further confirmed by sensitivity analysis demonstrating no significant change in the pooled ORs. Stratified analysis based on genotyping method did not modify the pooled result (Table 3.).

### *CD44* rs187115

**Figure 5:**
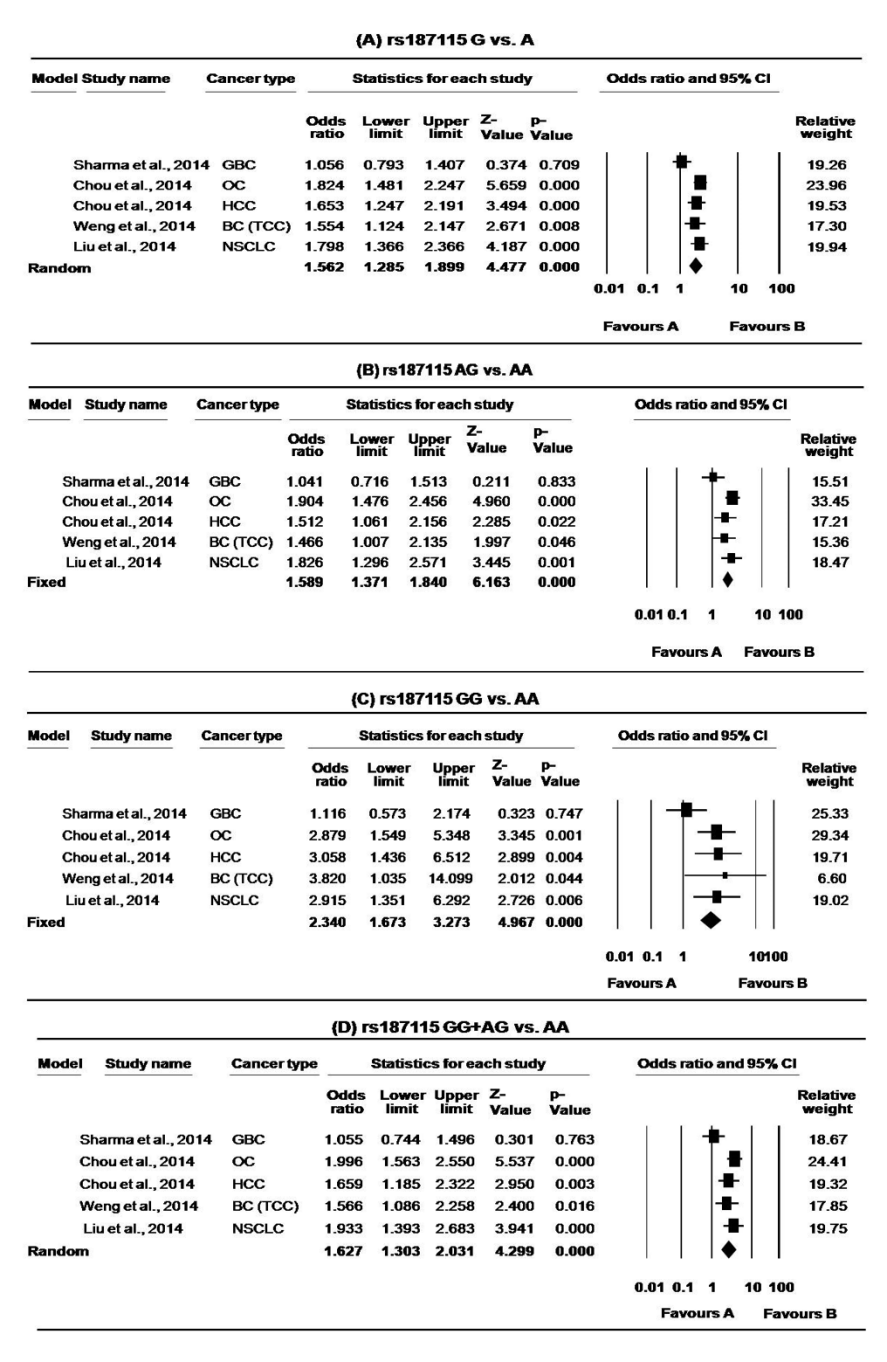
Forest plots for meta-analysis of *CD44* rs187115 polymorphism **A.** G *vs*. A **B.** AG *VS*. AA, **C.** GG *VS*. AA, **D.** GG + AG *VS.* AA and overall cancer risk. For each study, the estimates of OR and 95% CI were plotted with square and horizontal lines. The size of the square points is the relative weight of the respective study. Diamonds indicate the pooled OR and its 95% CI.

For rs187115, only six studies investigated the cancer risk, however the study of Verma et al. [47] deviated from HWE and hence was excluded. Thus, we included only five studies with 1716 cancer cases and 2065 controls for rs187115 meta-analysis [36, 38, 44-46]. The MAF for rs187115 ranged from 13 to 22%. Overall, individuals carrying the GG or AG genotype were at an increased risk of cancer compared with the AA genotype (GG *vs*. AA: OR = 2.34, 95% CI = 1.67-3.27, *p* = <0.000 and AG *vs*. AA: OR = 1.59, 95% CI = 1.37-1.84, *p* = <0.000, Table 3.). Moreover, significant associations were also found in G *vs*. A allele (OR = 1.56, 95% CI = 1.29-1.90, p = 0.000), as well as in dominant models (AG+GG *vs*. AA: OR = 1.63, 95% CI = 1.30-2.03, *p* = <0.000, Table 3., Figure 5.). The present meta-analysis revealed significant heterogeneity at the allele level (G *vs*. A: ph = 0.035, I^2^ = 61.402) and dominant model (AG+GG *vs*. AA: ph = 0.048, I^2^ = 58.222). Removal of study by Sharma et al. [44] was found to remove heterogeneity without affecting the overall result (G *vs*. A: ph = 0.837, I^2^ = 0.000; AG+GG *vs*. AA: ph = 0.656, I^2^ = 0.000. Due to limited number of studies, we could not perform stratified analysis for this SNP. Further, our sensitivity analysis confirmed the robustness of our findings.

### Publication bias

For rs13347, the review of funnel plot showed slight apparent asymmetry. Further, Egger's test as well as Begg and Mazumdar rank correlation tests did not demonstrate significant asymmetry except in the heterogenotype as well as dominant models (Table 4). However, stratified analysis based on study design and genotyping method did not revealed any significant biasness suggesting them as the main source of biasness in our meta-analysis (Figure 6). For other SNPs also, although funnel plot showed little asymmetry. Egger's as well as Begg and Mazumdar rank correlation tests demonstrated no apparent asymmetry except for rs11821102 in the variant genotype model (Table 4.). This may be because the number of studies is very low (5-7) to draw a more conclusive funnel plot

**Table 4:** Publication Bias for *CD44* Polymorphism

No. of studies	Case/ Control	V *vs*. W allele			VW *vs*. WW			VV *vs*. WW			VW+VV *vs*. WW		
		Egger's test		Begg's p2tailed	Egger's test		Begg's p2tailed	Egger's test		Begg's p2tailed	Egger's test		Begg's p2tailed
		T	P		T	p		T	p		t	p	
***CD44* rs13347**													
12	6612/7450	2.0295	0.0699	0.1148	2.7706	0.0198	0.0467	1.5961	0.1416	0.1926	2.5345	**0.0296**	0.0865
***CD44* rs11821102**													
7	3733/4454	0.8158	0.4517	1.0000	0.2789	0.7915	0.7639	5.2630	**0.003**	**0.0355**	0.2407	0.8194	1.0000
***CD44* rs10836347**													
7	4402/5167	0.5108	0.6313	0.7639	0.2645	0.8020	1.000	0.2551	0.8088	0.2296	0.2298	0.8274	0.7639
***CD44* rs713330**													
6	3453/3958	0.7957	0.4708	0.4524	0.0939	0.9297	0.7071	0.9319	0.4042	0.2597	0.3235	0.7626	0.7071
***CD44* rs187115**													
5	1716/2065	0.9790	0.3998	0.0864	1.8843	0.1560	0.0864	0.6987	0.5350	0.8065	1.4562	0.2414	0.0864

Significant associations are shown in bold. W- Wild allele, V- Variant allele

### Credibility of meta-analysis results

According to Venice guidelines, credibility of the cumulative association of CD44 variants with cancer risk are shown in Table 5. Our results demonstrated moderate evidence of association for CD44 rs13347 and rs187115 variants while weak evidence for rs11821102, rs10836347 and rs713330. This may be due to fewer number of studies as well as small sample sizes. Additionally, different genotyping methods and study designs contributing to likely biasness are the potential reasons for moderate or weak evidence of association.

**Table 5:** Credibility of the Association for CD44 Variants and Cancer Risk

Genetic variant	*Overall scheme	Cumulative evidence
CD44 rs13347	ABB	Moderate
CD44 rs11821102	CCA	Weak
CD44 rs10836347	CCA	Weak
CD44 rs713330	CCA	Weak
CD44 rs187115	BBB	Moderate

* First letter refers to the Amount of evidence that was assessed by counting the number of minor alleles. Grade A, B and C correspond if nminor =>1,000, 100-1000 and <100, respectively where nminor the total number of cases and controls with the least frequent genotype. Second letter refers replication assessment and the third letter demonstrated protection from bias [50].

## DISCUSSION

Single nucleotide polymorphism is the most common form of genetic variation, altering the expression level and/or function of any gene, thereby affecting an individuals' risk of cancer. In the present meta-analysis, we found that *CD44* SNPs significantly modulate the risk of cancer in Asians. Specifically, rs13347 and rs187115 were found to significantly increase the cancer risk while rs11821102 was associated with cancer protection. On the other hand, rs713330, rs10836347 did not affect an individual's susceptibility for cancer.

The rs13347C/T located in the 3′-untranslated region (UTR) of *CD44* is highly conserved and it is the main target region for microRNAs. The C to T base change of this SNP was found to disrupt the hsa-mir-509-3p binding site, thereby modifying the *CD44* mRNA stability and its expression. Further, functional studies established the association of T allele with enhanced transcriptional activity as compared with C allele [39, 48] and individuals carrying the T allele were shown to have higher expression of CD44 [42, 48]. In addition, it was reported to affect the hematopoietic stem cell mobilization in patients with hematologic malignancies.[51]. The rs13347C/T was significantly associated with an increased risk of CRC [48], NPC [39, 40], AML [41] and breast cancer [42], and this risk was found to increase as the number of variant alleles (T) increased. The rs13347T variant was also shown to be associated with tumor stage and lower five year survival rate in cancer patients [42, 48]. Although, some studies failed to find the association of rs13347 with various cancers [38, 43-46], our meta-analysis established that *CD44* rs13347 polymorphism is significantly associated with an overall increased risk of cancer. These findings suggest that this SNP may be used as potential biomarker for genetic susceptibility to various cancers in Asians.

The rs187115 SNP is located in the first intron of *CD44*. Intronic SNPs have been shown to play an important role in gene function by regulating its transcription and splicing [52]. Previously, this SNP was shown to be associated with cellular responses to a large panel of cytotoxic chemotherapeutic agents in a p53-dependent manner. In addition, the variant allele of this SNP was found to confer decreased drug sensitivity, poor overall survival and an earlier age of diagnosis in soft tissue sarcoma patients [35]. Further, several studies reported significant association of this SNP with increased susceptibility, development, invasion, advanced stage and poor prognosis of various cancers [38, 45, 46, 53]. Liu et al. (2014) reported that individuals having at least one copy of *CD44* rs187115 variant allele were associated with increased bone metastasis and tumor stage, as well as with decreased survival rate in NSCLC patients. Thus, this variant was suggested as a potential predictive marker of survival in NSCLC patients [38].

Though none of the studies demonstrated significant association of rs1182102 with cancer susceptibility, our result demonstrated a significant role of this SNP in cancer protection. The exact mechanism by which this SNP modulates cancer risk has not yet been elucidated; however, its location in the 3′UTR suggests that it alters the binding of miRNA contributing *CD44* deregulation. Further, our in-silico analysis also revealed the role of *CD44* rs1182102 in transcriptional regulation (Table 6.).

**Table 6:** Bio-informatics Analysis: Result of F-SNP

SNP	location	FS score	Functional category	Prediction tool	Prediction result
rs13347	3′UTR	0.176	Transcriptional regulation	TF search Golden Path	Changed Not exist
rs1182102	3′UTR	0.050	Transcriptional regulation	TF search Golden Path Consite	Not changed Not exist Changed
rs10836347	3′UTR	0.176	Transcriptional regulation	TF search Golden Path	Changed Not exist
rs713330	Intron	0.208	Transcriptional regulation	TF search Golden Path Consite	Changed Not exist Changed
rs187115	Intron	0.176	Transcriptional regulation	TF search Golden Path	Changed Not exist

To the best of our knowledge, we are the first to perform such a comprehensive meta-analysis of common functional polymorphisms of the *CD44* gene comprising all the published and well defined case-control studies. We followed a strict inclusion/exclusion criteria to avoid likely biases and NOS system was used to evaluate the quality of each studies demonstrating that all the included studies were of good (moderate to high) methodologic quality. In addition, our study has improved the statistical power of the analysis since we pooled large number of cases and controls from various studies. We also performed sensitivity analysis and multiple corrections to remove any false result, though the result remained unaffected, thereby adding weight to our findings. Since cancer is a highly fatal disease, our results investigating the association of functional SNP in *CD44* gene may have clinical significance in that they can help to identify interindividual differences in tumor susceptibility, recurrence capacity and chemoresistance among patients. However, care should be taken to interpret these results with caution as overall our study indicate moderate or weak evidence for association mainly due to limited number of studies.

### Study limitation

Though, we have collected all published articles till date, we could not perform a comprehensive subgroup analysis as the number of available studies were limited to Asian population and also for limited cancer types. In addition, there was significant heterogeneity for rs13347 meta-analysis, although in subgroup analysis it was removed or decreased Further, our results are based on unadjusted or crude estimates and the roles of haplotypes, gene-gene, and gene-environment interactions, as well as linkage disequilibriums were not considered. Last but not the least, we could not exclude the possibility of selection bias as study selection was limited to published results, articles in English language only and methodologies using different genotyping methods and study designs.

### Conclusions

We demonstrated a significant association of *CD44* SNPs in modulation of cancer risk. Specifically, rs13347 and rs187115 may be used as potential biomarkers for cancers in Asian populations. However, further analysis considering the aforementioned limitations and prognostic significance of *CD44* are required to better understand the role of these *CD44* SNPs in cancer risk.

## MATERIALS AND METHODS

### Literature search and study selection criteria

Following the PRISMA statement [54], we performed a systematic and comprehensive literature search on “Pubmed”, “Medline”, “Google Scholar”, “EMBASE”, and “Scopus” databases by using the following MeSH index keywords: “*CD44* gene”, “Cluster of differentiation”, in combination with “single nucleotide polymorphism (SNP) /variation/genotype”, and “cancer/carcinoma” or “tumor”. All published case-control studies investigating the association of *CD44* gene polymorphisms with human cancer susceptibility in English language were searched until May2016. All relevant studies were collected after thorough investigation of the abstracts of potential articles. Further, the reference lists of the selected articles and related reviews on the topic were manually examined to collect additional relevant studies.

The selection criteria of the studies were; original case-control study examining the association of *CD44* polymorphism with cancer risk having sufficient information to calculate the relative risk and 95% confidence intervals (CI), histo-pathologically confirmed cancer cases and healthy controls (free from any malignancy or other related pre-malignant condition such as benign and hyperplasia). On the other hand, studies unrelated to cancer research or lacking control population or sufficient data, and those not in accordance with Hardy-Weinberg equilibrium (HWE) in control groups were excluded from the meta-analysis. Duplicate or ecological studies, case reports, reviews, abstracts, comments and editorials were also excluded from the present meta-analysis.

### Data extraction

Two independent investigators separately weighed the eligibility of each study according to the inclusion and exclusion criteria listed above and any disagreements were further resolved by discussions and agreements. Data such as first author name, publication year, country of origin, ethnicity, genotyping methods, cancer types, frequency of cases and controls, genotype frequencies, minor allele frequencies, etc., were cautiously extracted from all eligible studies.

### Quality score assessment

The quality of each studies included in this meta-analysis (Ref) was rigorously evaluated independently by two authors (Rai Rajani and Gupta Usha), by using the Newcastle-Ottawa quality assessment scale (NOS) [55, 56] and all disagreements were resolved by discussion. The NOS is a star rating system in which each study was judged on standard criteria and subsequently categorized based on three fact: selection, comparability and exposure assessment with scores ranging from zero to nine stars. A study with NOS score of 7 to 9, 4 to 6 and 1 to 3 stars are usually considered to be a high, intermediate and low-methodological quality respectively.

### Statistical analysis

All statistical analyses were conducted using the Comprehensive Meta-analysis software (Version 2.0, BIOSTAT, Englewood, NJ). The pooled ORs were estimated for allele contrast, log-additive and dominant models. Odd's ratio greater than 1 is considered significant. Heterogeneity was measured using the I^2^ value and Chi-square-based Q statistics (significant at *p* < 0.05). I^2^ = 0%, 25%, 50% and 75% were considered as no, low, moderate, and high observed heterogeneity, respectively [57]. In the case of significant heterogeneity, the random-effect model was used to calculate the pooled ORs [58, 59]. Funnel plot and Egger tests were performed to examine the publication bias [60]. Moreover, sensitivity analysis was performed to check if alteration of the inclusion criteria affects the results of the meta-analysis. To adjust the p values for multiple comparisons in subgroup analyses, we applied the Benjamini-Hochberg (BH) step-up correction method, which control the false discovery rate (FDR) yielding p_corr_. A p_corr_value less than 0.05 was considered as significant [61]. Hardy-Weinberg equilibrium (HWE) test of SNP was performed using Michael H. Court's (2005-2008) online calculator (http://www.tufts.edu/~mcourt01/Documents/Court%20lab%20-%20HW%20calculator.xls). Further, in-silico study was performed by using online Web servers- FastsnP (http://fastsnp.ibms.sinica.edu.tw) and F-SNP (http://compbio.cs.queensu.ca/F-snP/) to predict the functional effect of each SNPs.

### Credibility of meta-analysis results

The credibility of the cumulative association of CD44 polymorphisms and the cancer risk was scrutinized by using Venice interim criteria [50] including a set of three scores (the amount of evidence, replication of results, and protection from bias) which are used to grade the evidence produced by the study. Each of these three scores can attain a maximum of ‘A’ grade, followed by ‘B’, and ‘C’. Finally, the grades may be scored as follows- strong evidence (AAA), moderate evidence (AAB, ABA, ABB, BAA, BBA, BBB, BAB) and weak evidence (rest all scores).
